# Prognostic stratification improvement by integrating ID1/ID3/IGJ gene expression signature and immunophenotypic profile in adult patients with B-ALL

**DOI:** 10.1186/s13046-017-0506-4

**Published:** 2017-02-28

**Authors:** Nataly Cruz-Rodriguez, Alba L. Combita, Leonardo J. Enciso, Lauren F. Raney, Paula L. Pinzon, Olga C. Lozano, Alba M. Campos, Niyireth Peñaloza, Julio Solano, Maria V. Herrera, Jovanny Zabaleta, Sandra Quijano

**Affiliations:** 1 Programa de Investigación e Innovación en Leucemias Agudas y Crónicas (PILAC), Instituto Nacional de Cancerología, Bogotá, Colombia; 2 Grupo de Investigación en Biología del Cáncer, Instituto Nacional de Cancerología, Bogotá, Colombia; 3 Programa de Doctorado en Ciencias Biológicas, Pontificia Universidad Javeriana, Bogotá, Colombia; 4 Facultad de Medicina, Universidad Nacional de Colombia, Bogotá, Colombia; 5 Grupo de Hemato-Oncología, Instituto Nacional de Cancerología, Bogotá, Colombia; 6 Department of Pediatrics, Pediatric Hematology-Oncology Louisiana State University Health Sciences Center, New Orleans, LA USA; 7 Children’s Hospital of New Orleans, New Orleans, LA USA; 8 Hospital Universitario San Ignacio, Bogotá, Colombia; 9 Department of Pediatrics, Louisiana State University Health Sciences Center, New Orleans, LA USA; 10 Stanley S. Scott Cancer Center, Louisiana State University Health Sciences Center, New Orleans, LA USA; 11 Grupo de Inmunobiología y Biología Celular, Departamento de Microbiología, Facultad de Ciencias, Pontificia Universidad Javeriana, Bogotá, Colombia

**Keywords:** B-ALL, Risk-stratification, Survival, Gene-expression, Immunophenotype

## Abstract

**Background:**

Survival of adults with B-Acute Lymphoblastic Leukemia requires accurate risk stratification of patients in order to provide the appropriate therapy. Contemporary techniques, using clinical and cytogenetic variables are incomplete for prognosis prediction.

**Methods:**

To improve the classification of adult patients diagnosed with B-ALL into prognosis groups, two strategies were examined and combined: the expression of the *ID1/ID3/IGJ* gene signature by RT-PCR and the immunophenotypic profile of 19 markers proposed in the EuroFlow protocol by Flow Cytometry in bone marrow samples.

**Results:**

Both techniques were correlated to stratify patients into prognostic groups. An inverse relationship between survival and expression of the three-genes signature was observed and an immunophenotypic profile associated with clinical outcome was identified. Markers CD10 and CD20 were correlated with simultaneous overexpression of *ID1, ID3* and *IGJ.* Patients with simultaneous expression of the poor prognosis gene signature and overexpression of CD10 or CD20, had worse Event Free Survival and Overall Survival than patients who had either the poor prognosis gene expression signature or only CD20 or CD10 overexpressed.

**Conclusion:**

By utilizing the combined evaluation of these two immunophenotypic markers along with the poor prognosis gene expression signature, the risk stratification can be significantly strengthened. Further studies including a large number of patients are needed to confirm these findings.

**Electronic supplementary material:**

The online version of this article (doi:10.1186/s13046-017-0506-4) contains supplementary material, which is available to authorized users.

## Background

B-ALL is a malignant hematological disorder, with heterogeneous clinical, cellular and molecular characteristics, response to therapy and risk of relapse [1, 2]. Biologically, significant advances in the identification and molecular characterization of genetic alterations that have an important role in the biology and evolution of B-ALL have been described [3]. In adult patients, B-ALL has a poor clinical outcome and low survival rates with an estimated rate of complete remission (CR) of 75% and disease-free survival (DFS) lower than 30% [4–7]. Currently, the criteria used to stratify patients into risk groups at diagnosis is based on a range of variables that include age at diagnosis, white blood cell count (WBCC), and cytogenetic and chromosomal alterations [8, 9]. However, applying these variables for risk assessment may be inadequate because some patients initially classified as standard risk group with favorable prognostic features, may experience treatment failure, relapse and/or death during the course of their disease [10]. Therefore, it is necessary to implement new and better strategies to be able to properly classify patients into risk groups from the time of diagnosis, in order to minimize the risk of both relapse of disease and death by toxicity to drugs caused by overtreatment.

The analysis of acute leukemia by Flow Cytometry (FCM), performed in all patients at diagnosis, is of great significance in routine clinical practice for the classification and monitoring of diseases in conjunction with morphological cytogenetic and molecular analysis [11]. Immunophenotyping has provided relevant information for the diagnosis, classification and monitoring of hematological malignancies [12]. Consequently, the assessment of immunophenoype by FCM has become essential and is part of the current World Health Organization (WHO) classification of hematological malignancies.

In 2006 the European Union-supported EuroFlow Consortium (EU-FP6, LSHB-CT-2006-018708) started a project aimed at the prospective design and evaluation of panels of antibodies for the diagnosis and classification of the most frequent subtypes of leukemias and lymphomas, in which immunophenotyping has proven to be relevant [13]. Particularly, in the study of acute leukemias EuroFlow has contributed in the development of highly sensitive and standardized FCM. These protocols describe the optimal antibody panels, the design and evaluation of adequate standard operating procedures (SOPs) for instrument setup, fluorescence compensation and sample preparation and elaboration of adequate software tools for the overall evaluation of the phenotypic profiles obtained [13]. These advances have significantly improved detection of Minimal Residual Disease (MRD), which assesses response to therapy, and is an important prognostic indicator.

Another useful tool that has improved the understanding of the pathogenesis and cell biology of acute leukemias is the detection of chromosomal rearrangements. Various studies have shown that chromosomal rearrangements, compared with other molecular techniques, can determine the prognosis of the disease more accurately [8, 9]. In addition, while many isolated chromosomal rearrangements are not able to induce leukemia in experimental models on their own, the development of high-resolution profiles of genetic alterations have further improved the understanding of the genetic basis of this disease. However, in approximately 50% of B-ALL cases these chromosomal abnormalities are not observed, suggesting that additional submicroscopic genetic alterations contribute to leukemogenesis [10].

For over a decade the identification of numerical alterations of DNA (gain and loss) has been possible through molecular techniques such as sequencing and microarrays [14]. These alterations, which are different from translocations, can define new disease subtypes and affect the response to treatment. Similarly, these alterations may constitute groups of genetic alterations that together contribute to the establishment and persistence of malignant clones and clonal evolution of tumor cells [15–20]. Many of the altered genes encode regulators of lymphoid development, cell cycle, tumor suppressors or lymphoid signaling molecules [16, 21].

Some studies using DNA microarray technology are in progress in the hopes of identifying novel markers of relapse in this patient subgroup. We have recently described, for the first time, a 3-gene signature, *ID1/ID3/IGJ*, that when highly expressed constitutes a poor prognostic factor in B-ALL adult patients with impact in low rates of CR, poor OS and shorter EFS [22]. ID family genes (as *ID1* and *ID3*) are transcription factors, inhibitors of differentiation, and proliferation and cell cycle regulators [23]. ID genes have been associated with the biology and pathogenesis of various cancer models, including breast, brain, colorectal, prostate, ovary, liver and pancreas [23, 24]. We hypothesize that our 3-gene signature could be implemented in routine diagnostic evaluation to make a more accurate classification of patients into risk groups. In this work we assessed the relationship between the molecular signature *ID1/ID3/IGJ* and the EuroFlow immunophenotypic features, to determine the prognosis of patients at the diagnosis in a comprehensive and accurate way.

## Methods

### Patients and samples

Forty-one bone marrow samples and two peripheral blood samples obtained at diagnosis from 43 B-ALL adult patients (19 women and 24 men; median age 30; age range 16–63 years old) were analyzed in this study. All the individuals provided written informed consent prior to enrolling in the study, and the study was approved by the Ethics Committees of the participating centers (Instituto Nacional de Cancerología and Hospital Universitario San Ignacio, Bogotá, Colombia). As inclusion criteria, all patients were older than 15 years who had not received previous chemotherapy treatment. The diagnosis of B-ALL was confirmed by myelogram analysis in bone marrow and biopsy aspirates and by immunophenotypic analysis by FCM. The average percentage of tumor infiltration in bone marrow, detected by FCM, was 82% (range 20–95%) and in peripheral blood was 189 blasts/uL (range 0–210600). Samples of normal bone marrow from three patients with diagnosis of solid tumors in CR, without infiltration of bone marrow and therefore without hematologic abnormalities, were used as control.

### Gene expression analysis by Real Time PCR

This analysis was performed as we have previously reported [22]. Briefly, the gene expression signature was validated by RT-PCR using TaqMan probes to quantify the expression levels of *ID1, ID3* and *IGJ* mRNA. The reaction was amplified in a QuantStudio 12 K plex Real-Time PCR machine (Applied Biosystems). The 2^-ΔΔCT^ method was used to estimate the fold induction of each gene using *GAPDH* and an internal calibrator as controls. RT-PCR assays were done in triplicate.

### Immunophenotyping analysis by flow cytomety

Immunophenotype analysis for all samples was performed using the panel of antibodies recommended and standardized by the European consortium EuroFlow [12]. The B lymphoblasts phenotype was determined using antibody combinations in eight different fluorescences (V450, V500c, FITC, PE, PerCpCy5.5, PECy7, APC, APCH7). The immunophenotyping panel included backbone markers present in all tubes, allowing the identification of the blast population (CD34, CD19 and CD45), characterization markers useful to distinguish B- development patterns (CD20, CD10, kappa, lambda, SmIgM, cyIgM, CD24, CD22, nTdT, CD58, CD9 and CD38), myeloid-lineage associated markers, which can be expressed aberrantly in >80% of B-ALL cases (CD66, CD13, CD33, CD117) [12, 25]. After the labeling, the samples were acquired on a BDB FACSCanto II flow cytometer, using the FACSDiva (BDB) software program. Fifty thousand events were collected per sample. Data analysis was performed with the program INFINICYT TM (Cytognos SL, Salamanca, Spain) to determine the expression level and mean fluorescence intensity (MFI) for each of the antigens in the cells. Cell populations were classified as positive for each marker if the expression (percentage and MFI) was higher than that was observed in the negative control (basal autofluorescence basal cells without antibody). In addition, the MFI of each marker in leukemic populations was compared to the MFI of the same marker in normal bone marrow B-cell populations (normal counterpart) to define whether the analyzed marker was negative, under-expressed or overexpressed on the blast population [26]. The panel of 19 markers was evaluated in 42 patient samples at baseline to confirm the diagnosis of B-ALL and after induction chemotherapy to detect the presence of disease (MRD). Instrument setup, calibration and quality control were performed during the study using standard commercial reagents (BD Cytometer Setup and Tracking Beads and BD Comp Beads; BD), according to the manufacturer’s instructions.

### Statistical analysis

Clustering analyses and heatmaps were performed using R-project (www.r-project.org), GenomeStudio (Illumina) and Gene set Enrichment Analysis (GSEA, http://software.broadinstitute.org/gsea/index.jsp). Statistical analysis was performed using SPSS software (version 22 for windows) and R. *p*-values that were <0.05 were considered statistically significant. Survival analyses were estimated by Kaplan-Meier curves and differences between the survival functions were assessed with the log-rank test. GraphPad graphic software was used to plot the immunophenotype markers expression data.

## Results

### *ID1/ID3/IGJ* gene expression signature is a variable with high prognostic impact in adult B-ALL patients

**Fig. 1:**
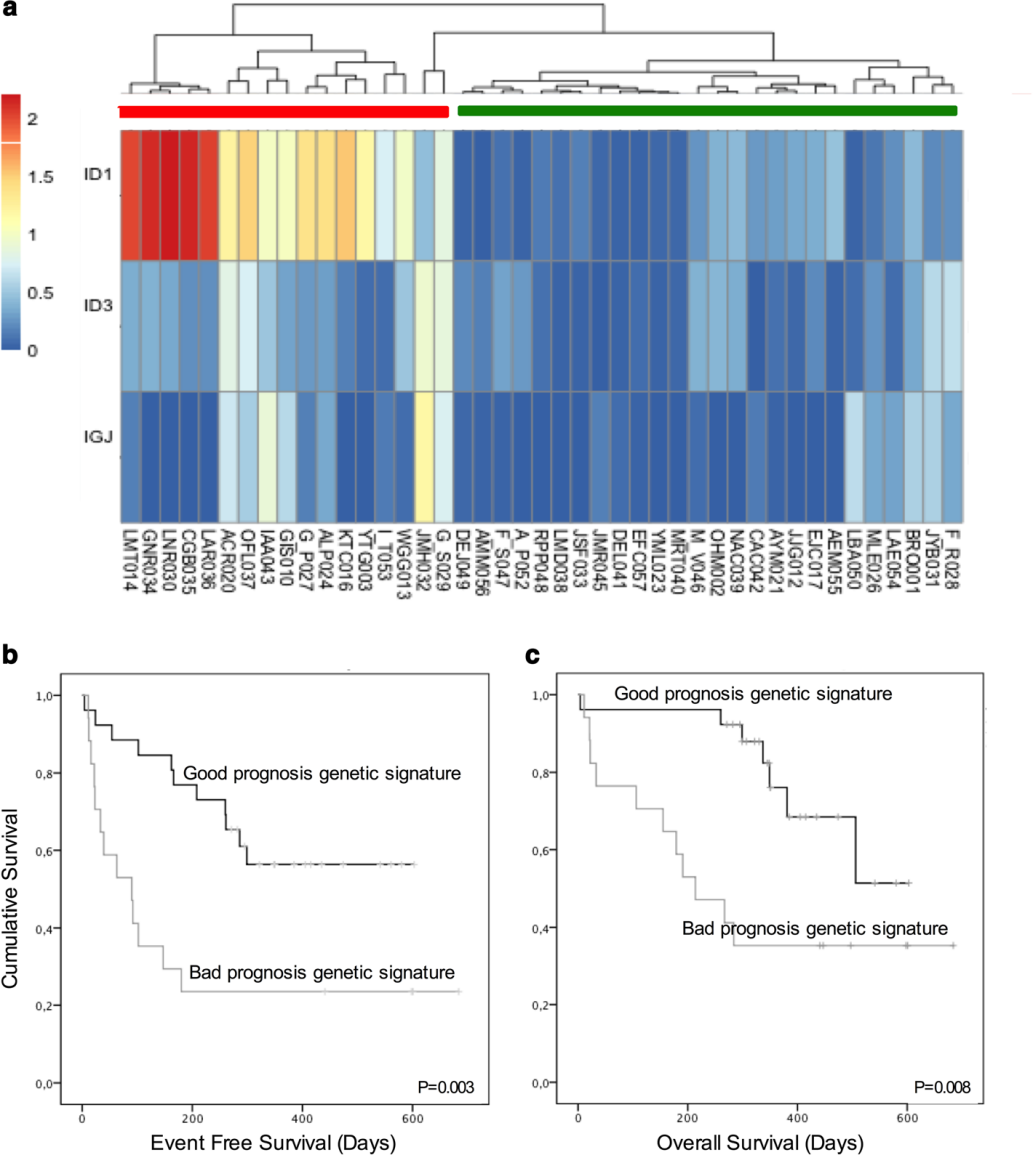
Gene expression signature predictor of prognosis. **a** Unsupervised cluster analysis applied to 43 patients with B-ALL according to the expression of our 3-genes signature predictor of prognostic. Cluster analysis discriminates 2 different groups (*Green bar good prognosis group and red bar poor prognosis group*). Kaplan Meier curves for event-free survival (*p* = 0.001) **b** and overall survival (*p* = 0.001) **c** according to the presence of 3-genes signature of poor prognosis (*red curve*) vs patients without the expression profile of poor prognosis (*green curve*). The expression profile of poor prognosis is defined as the simultaneous overexpression of genes ID1, ID3 and IGJ

To analyze the prognostic relevance of molecular signatures defined by gene expression profiling in adult B-ALL, we analyzed 43 primary leukemia samples using gene expression microarrays and confirmed the most relevant findings with RT-PCR [22]. Unsupervised clustering analysis according to the three most differentially expressed genes (*ID1, ID3*, and *IGJ*) between responders and non-responders, revealed the presence of two robust clusters of samples with different gene expression profiles, and with different clinical characteristics. The first cluster (green bar in Fig. 1a), was characterized by a gene expression signature related to a good prognosis: younger patients (<30 years old), lower WBCC (<30.000/ul), and lower tumor load at diagnosis in both bone marrow and peripheral blood at the time of diagnosis; in addition, 94% of the patients in this group achieved CR. In contrast, the second cluster, referred here as a predicted poor prognosis group (red bar in Fig. 1a) included 40% of patients with failure to induction therapy and was associated with worse prognostic variables. Notably, the simultaneous over-expression of ID1, ID3 and IGJ genes was found as an independent prognostic feature that identifies B-ALL patients with poor EFS (*p* = 0.001, Fig. 1b) and OS (*p* = 0.001, Fig. 1c).

### Comparative analysis of immunophenotype of normal and leukemic cells

Similar to what has been reported in the literature, comparative immunophenotype analysis between B-ALL cases and their normal counterpart, revealed high heterogeneity in the expression of the different markers suggesting the existence of leukemia associated phenotypes (LAP) [11, 26] in our cohort of patients. Leukemic lymphoblast cells were characterized by over-expression (median fluorescence channel) of CD34 and CD10 immaturity markers, B lineage markers such as CD19 and CD24, the lymphocyte common antigen CD45 and aberrant expression of myeloid lineage associated marker CD66. Additionally, blast cells showed higher light scatter characteristics (Forward-Scatter –FSC- and Side-Scatter –SSC-). Finally, an under-expression of CD38, CD117, CD9 and TdT was observed B-ALL cells, as compared to normal B-cell precursors (Additional file 1).

### Immunophenotype profile defines risk groups and has significant prognosis value

**Fig. 2:**
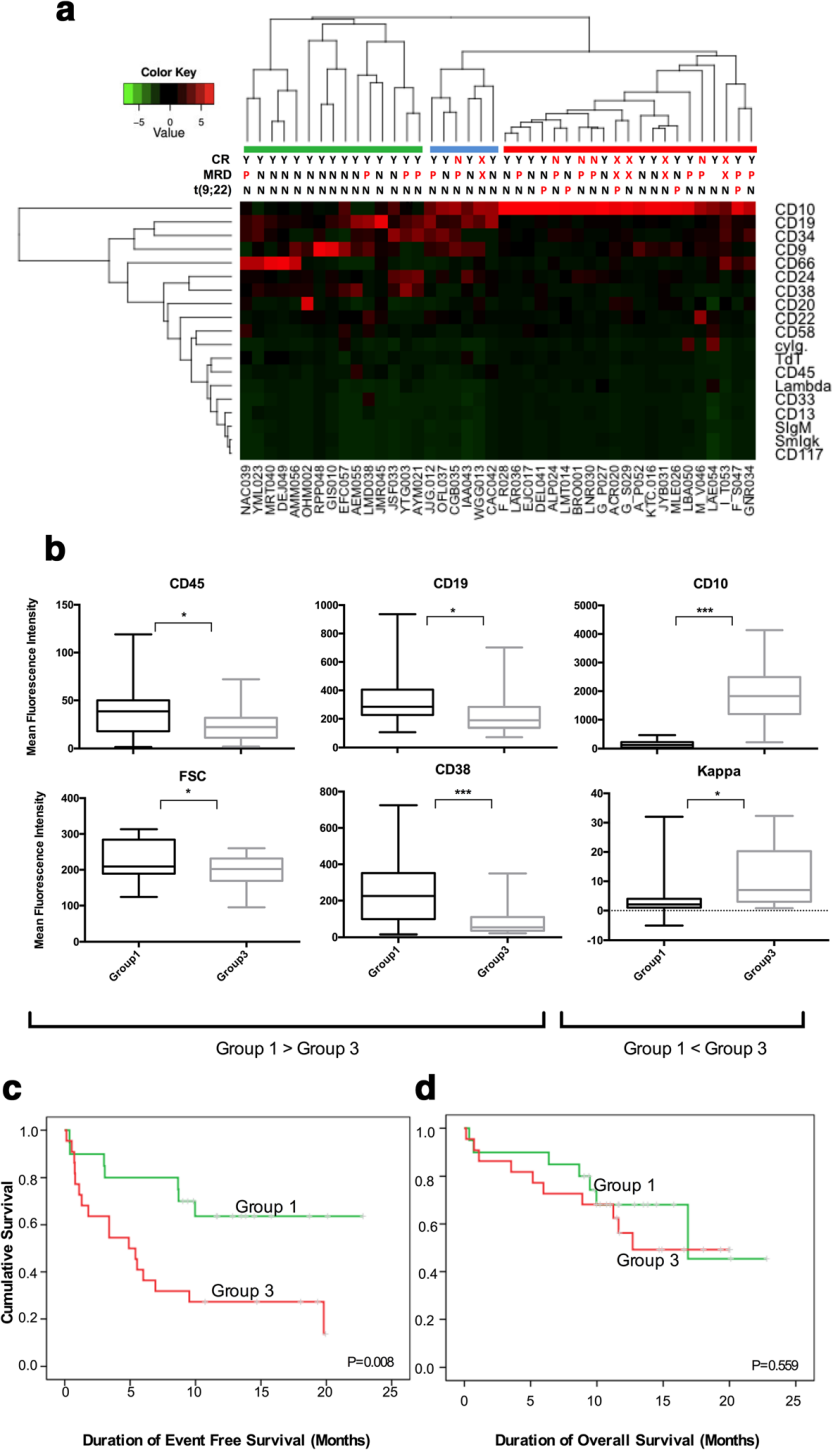
Determination of groups with differential immunophenotypic markers expression. **a** Unsupervised hierarchical clustering analysis in diagnosis bone marrow of B-LLA patients according to the expression of the evaluated immunophenotypic markers revealed three main groups of patients. *Green bar* (group 1) corresponds to the group with high rates of complete remission, low number of patients with positive MRD and absence of patients with t (9; 22). While *red bar* (group 3) represents the group of patients with the highest percentage of induction failures, positive MRD and all patients with t(9; 22). *Blue bar* (group 2) represents a small group of patients with intermediate outcome compared with the two groups in the extremes. CR: complete remission. MRD: minimal residual disease. **b** Expression levels of immunophenotypic markers in the two groups formed at the ends of the heatmap with different clinical features (groups 1 and 3). CD45, CD19, CD38 and FSC have lower expression in group 3 than in group 1. CD10 and Kappa have different high expression in group 3 compared to group 1. The differences are significant (*p = <0.05, ***p = <0.0001). Kaplan Meier curves for event-free survival (**c**) (*p* = 0.008) and overall survival (**d**) (*p* = 0.559) in the two prognostic groups identified by immunophenotypic profile. Note that EFS curves showed statistically significant differences, whereas the curves for OS did not

To analyze the prognostic relevance of the 19 EuroFlow immunophenotype markers we used the value of mean fluorescence channel in combination with light scattering parameters (FSC and SSC) in the samples of our 42 patients with adult B-ALL. Unsupervised clustering analysis, based on the immunophenotype, revealed the presence of three clusters of samples (Fig. 2a). The first cluster (green bar) was a group characterized by an immunophenotype signature associated with a better outcome of the disease in which all patients (*n* = 14) achieved CR, only 3 (21%) presented positive MRD and none of them presented chromosomal rearrangements at diagnosis or died during induction treatment. A second cluster, constituted by 21 samples from patients (red bar) with an immunophenotype profile associated with a poor prognosis, including 4 (19%) with induction treatment failure, 8 (38%) with MRD positive and four who died during the induction phase. It is noteworthy that all patients with t(9;22) + included in our cohort (*n* = 5) were included in this group. A third group of samples (blue bar), represents a small group of six patients with intermediate outcome compared with the two extreme groups. In this group, one patient failed to achieve CR and one more died during induction therapy. Two patients had positive MRD and none of them had t(9; 22) + .

Comparison of clinical characteristics between the groups represented by the green and red bars (Group 1 and Group 3, respectively) showed that patients were significantly different in age (median 25 vs 38 years, respectively). Additionally, WBCC at diagnosis was higher in group 3, although no statistically significant difference was found (median: 8830 vs 12,860 leucocytes/μl respectively) (Additional file 2). When we analyze the expression level of immunophenotypic markers, a statistically significant higher level of CD10 expression and reduced expression of CD19, CD38 and CD45 was observed in the poor prognosis group (Group 3) compared with patients with favorable outcome (Group 1) (Fig. 2b). Non-significant increased expression of CD20, CD66 and CD117 markers were also observed in the group with worse outcome (Additional file 3).

Moreover, survival analysis considering the immunophenotypic profile, revealed that over-expression of CD10 and under-expression of CD19, CD45 and CD38 were associated with a poor EFS (Fig. 2c) without impact on OS (Fig. 2d).

### Gene expression signature is associated with the immunophenotypical prognostic profile of B-ALL

**Fig. 3:**
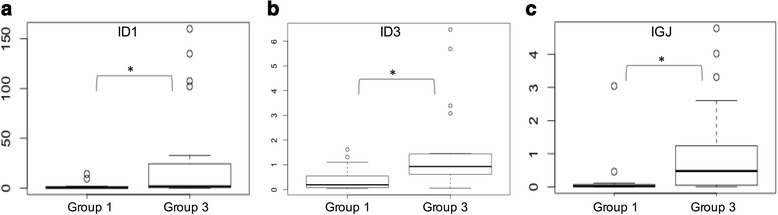
Expression levels of genes **a** ID1 (*p* = 0.0432), **b** ID3 (*p* = 0.024) and **c** IGJ (*p* = 0.0399) included in the poor prognosis genetic signature in groups determined by immunophenotypic expression pattern

We then determined whether there was an association between the identified gene expression signature (*ID1, ID3, IGJ*) with the immunophenotypic expression. We found that patients in group 1 showed significantly lower levels of expression of the 3 genes (*ID1 p* = 0.0432, *ID3 p* = 0.024, *IGJ p* = 0.0399), when compared with patients belonging to group 3 defined by the expression of poor prognosis immunophenotypic markers (Fig. 3a, b, and c). A correlation analysis was performed to determine if there was an association between these two molecular signatures (gene expression and immunophenotype). As can be seen in Table 1, a significant positive correlation between high expression of *ID1* and lambda and kappa light chains, as well as a significant negative correlations with CD38 expression was found. Overexpression of *ID3* gene correlated with decreased in CD38 expression and greater complexity of tumor cells (SSC). High expression of *IGJ* also correlated with elevated expression of lambda chains. The simultaneous overexpression of the gene signature *ID1, ID3* and *IGJ* was correlated with elevated expression of markers associated with B-cells differentiation and maturation B (CD20, CD10. *p*-values in boldface, Table 1). It is interesting to note that most of the immunophenotypic markers that correlate with the poor prognosis gene signature are markers that allow the separation into different prognostic groups.

**Table 1:** Correlation of immunophenotyping markers and gene expression signature ID1/ID3/IGJ

	CD20		CD10		Lambda		CD38		Slgmk		SSC	
	Spearman R	*p*-value	Spearman R	*p*-value	Spearman R	*p*-value	Spearman R	*p*-value	Spearman R	*p*-value	Spearman R	*p*-value
ID3	0.512	**0.001**	0.454	**0.002**	0.292	0.06	−0.318	0.04	0.258	0.09	0.343	0.02
ID1	0.298	**0.05**	0.317	**0.04**	0.361	0.01	−0.345	0.02	0.358	0.02	0.291	0.06
IGJ	0.337	**0.02**	0.337	**0.01**	0.337	0.02	−0.181	0.250	0.178	0.2592	0.121	0.442

### Clustering analysis defined by immunophenotypic markers that correlate with gene signature ID1/ID3/IGJ defines B-ALL patients with poor prognosis

**Fig. 4:**
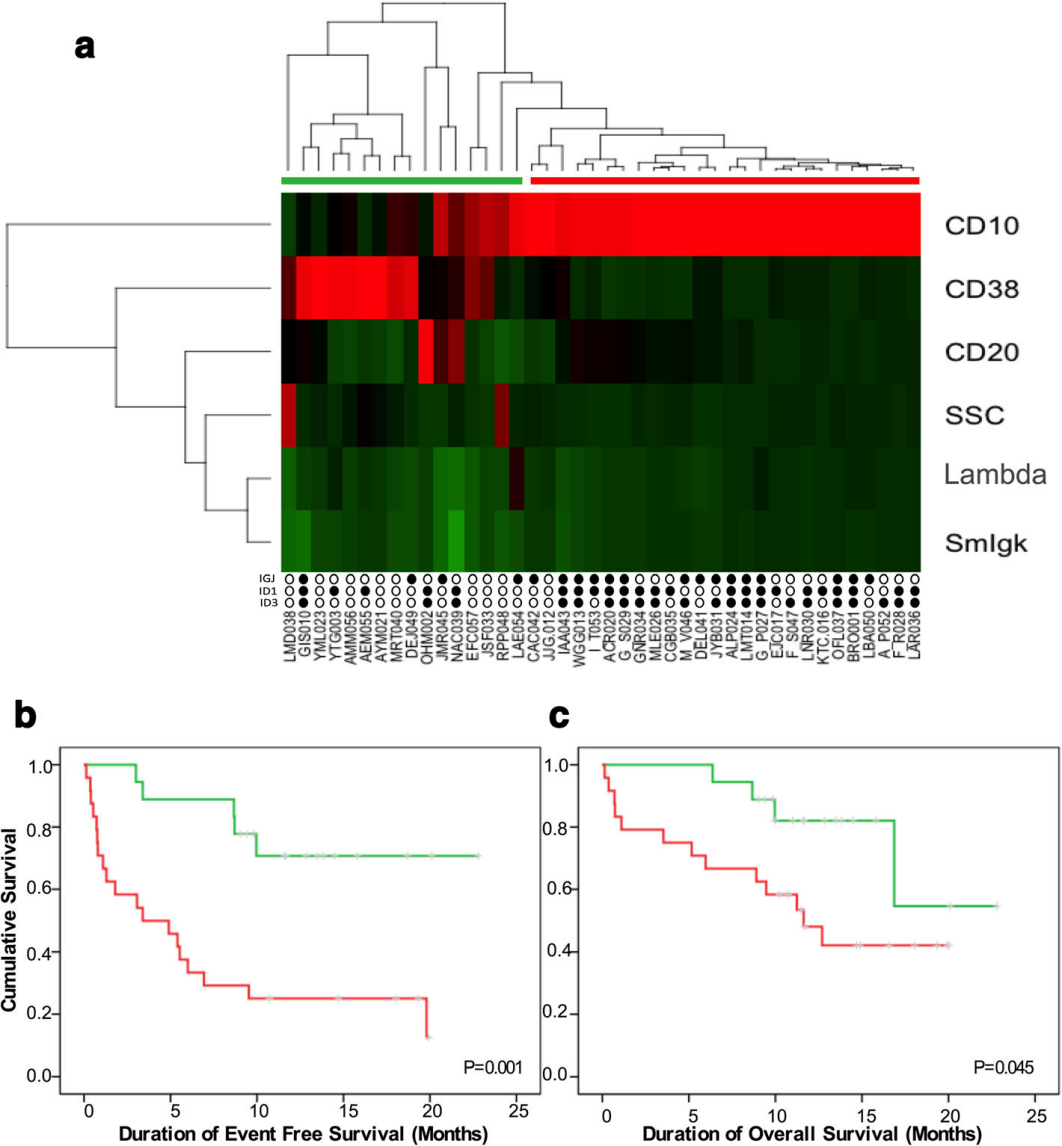
**a** Heatmap and clustering analysis according to immunophenotypic markers expression correlated with poor prognosis genetic signature. Note that at the *bottom* of heatmap it is shown that patients in *green group* are those with *lower* altered expression of ID1, ID3, IGJ genes. While most patients with overexpression of two or more of these genes are included in the *red* group. Kaplan-Meier curves for event-free survival (*p* = 0.001) **b** and overall survival (*p* = 0.045) **c** according to the groups defined by the heatmap in **a**

Using an unsupervised hierarchical clustering analysis based on the expression of immunophenotypic markers that are most correlated with our gene expression signature, we observed two groups with differential immunophenotypic profile. The first group (red bar in Fig. 4a) included patients with poor response to treatment, and high rates of positive MRD, presented with elevated expression of CD10, CD20, Lambda and Kappa proteins and increased in cellular complexity, as well as decreased CD38 expression. This group, with poor prognostic features, was closely associated with gene expression status. As can be seen at the bottom of Fig. 4a, this group included most of the patients with at least 2 of the genes overexpressed, suggesting a molecular signature of immaturity and maturational dyssynchrony.

Analysis of the prognosis of these two different groups revealed that the group with immunophenotypic and genetic characteristics of poor prognosis (red bar) had both lower event-free survival (Fig. 4b, red line) and lower overall survival (Fig. 4c) than the group with good prognostic features (green line). As shown in Fig. 4, statistically significant differences for both EFS and OS (*p* = 0.001 and *p* = 0.045) were found between the two groups. However, as noted, the difference was much greater in EFS. Nevertheless, it is important to highlight that OS curves were better discriminated once the immunophenotypic and genetic characteristics were integrated (Fig. 4c) than when calculated only accounting for the immunophenotypic features (Figs. 4c and 2d).

### Integration of gene expression signature ID1/ID3/IGJ together with the over-expression of CD10 and CD20 improves stratification of patients in terms of survival

**Fig. 5:**
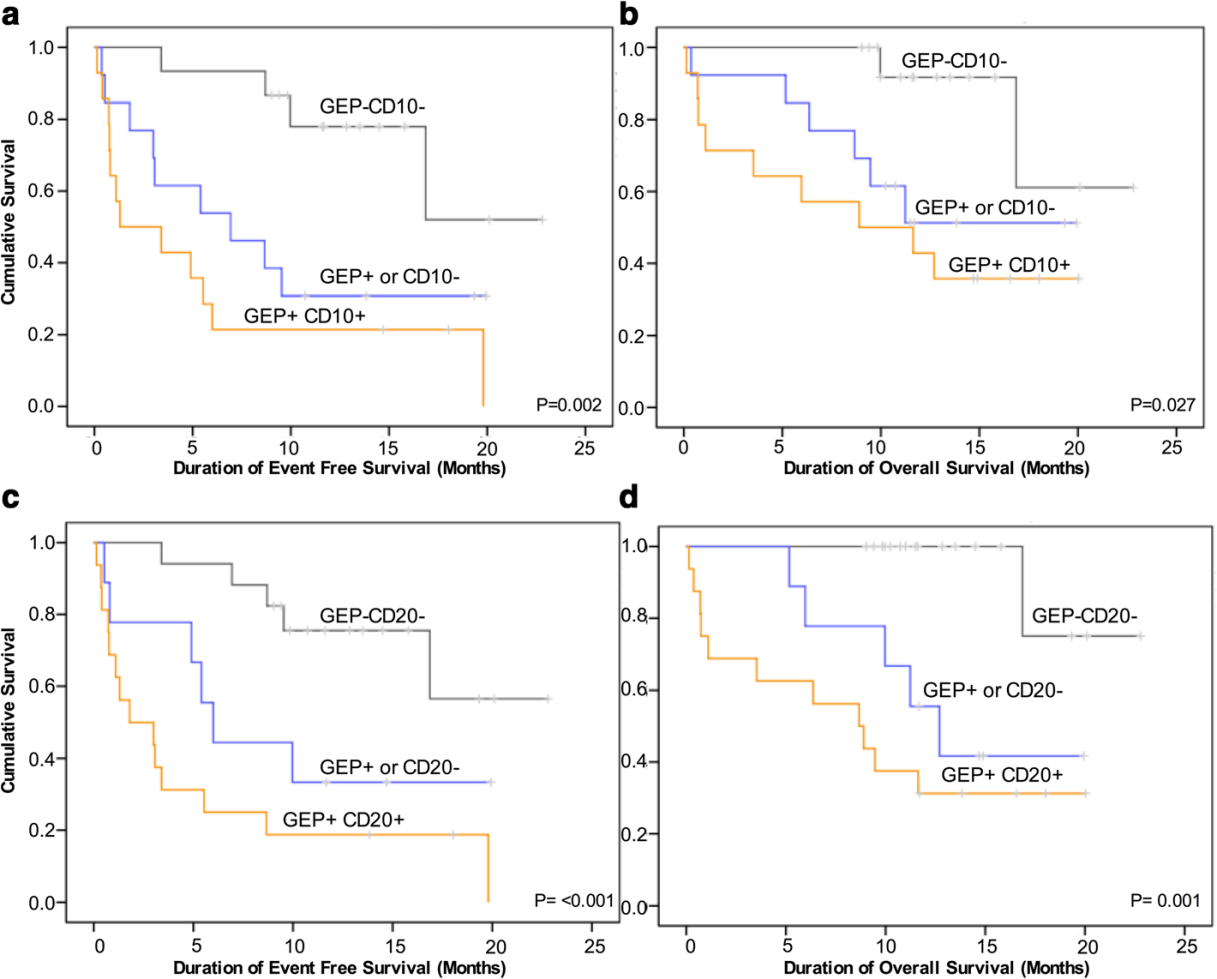
Event free survival (**a**, **c**) and overall survival (**b**, **d**) of 42 patients according to the categorization defined by the presence of the genetic signature and CD10 or CD20 expression. *p* values correspond to differences between *gray* and *yellow* curves

Due to the significant interaction between differentiation and maturation markers (CD10, CD20) with gene expression signature (Table 1), we then categorized patient molecular characteristics into 3 groups, based on the expression of the gene signature and the maturation markers: 1) Absence of both poor prognosis gene expression signature and absence of CD10 over-expression (GEP-/CD10-); 2) isolated presence of either poor prognosis gene expression signature or CD10 over-expression (GEP+ or CD10+), in the absence of the other; and 3) simultaneous presence of both poor prognosis gene expression signature and CD10 overexpression (GEP+/CD10+). The GEP-/ CD10- group showed the best OS and EFS while the GEP+/CD10+ had the worst (Fig. 5a and b, respectively). Interestingly, the GEP+ or CD10+ showed intermediate values for both parameters (Fig. 5a and b). Likewise, the same findings were obtained when the analysis was done with CD20 expression (Fig. 5c and d).

## Discussion and conclusions

The current multi-variable risk classification strategies are unable to adequately stratify patients into risk groups from the time of diagnosis. Thus, the implementation of a stronger risk stratification tool should help minimize both the risk of relapse of disease and death by toxicity of B-ALL patients. Recently, we identified a signature of three genes (*ID1, ID3* and *IGJ*), whose simultaneous over-expression confers a worse prognosis with reduced EFS and OS in B-ALL adult patients [22] (Fig. 1). In this study, we wanted to evaluate the role of this gene signature in combination with EuroFlow immunophenotypic B-ALL panel as a prognostic tool in adult patients with B-ALL.

Although Immunophenotyping has provided relevant information for the diagnosis, classification and monitoring of hematological malignancies, there is scarce information about its use as a prognostic parameter. Initially, we evaluated the ability of immunophenotype to predict prognosis in B-ALL. We found that the immunophehnotype correlates with clinical features and outcome. Individuals who had tumoral lymphoblasts with lower expression of CD45, CD19, CD38 and high expression of CD10, CD20 and kappa are patients with poor prognosis and low EFS. This group of patients had low CR rates, and a high percentage of MRD+. Importantly, this group included all patients with t(9;22). Additionally, they have a higher WBCC and median age over 30 years.

The expression of *ID1/ID3/IGJ* was higher in the group with the poor prognosis immunophenotype profile and a statistically significant correlation between the expression of these 3 genes with high expression of CD10 and CD20 was found. Clustering analysis according to the immunophenotypic markers correlated with the gene expression signature, suggesting that patients with both poor prognosis gene expression signature and overexpression of CD10 or CD20, had worse EFS and worse OS than patients with either one of the profiles.

In general, studies assessing the gene expression profiles in B-ALL adult patients are scarce. However, similar to our results, several studies have experimentally addressed the idea that ID (*ID1, ID2, ID3* and *ID4*) proteins are markers for prognosis and possible therapeutic targets in cancer. They have found that high levels of gene expression, and consequently high levels of ID proteins, appear to drive certain human tumors. As a result, the elevated ID1 and ID3 expression have been associated with worse prognosis in bladder, breast, brain, colon, rectum, stomach, kidney, pancreas, prostate, ovary and liver tumors and also in acute myeloid leukemia [27–37]. In contrast, few studies have associated the elevated ID1 and ID3 expression with good prognosis in models of breast and brain cancer [38, 39]. In turn, the *IGJ* gene, which was also found over-expressed in our group with poor response to induction therapy, has been reported as part of a poor prognosis signature in B-ALL pediatric patients; interestingly, they had high Hispanic/Latino ethnicity, low 4-years event-free survival and high frequency of positive MRD [40].

Our findings demonstrate for the first time, that in addition to serving as a diagnostic tool, the immunophenotypic panel for B-ALL standardized by EuroFlow, may also have implications for better prognostic classification of patients, especially when used association with the gene expression profile of tumor cells. In this regard, the differential analysis of immunophenotype shows that there is heterogeneity in the expression of different markers evaluated and these are associated with the survival of patients and with the poor prognosis gene expression signature. The presence of CD38 (>30%) is associated with worse prognosis in mature B-cell tumors like B-chronic lymphoid leukemia and in hairy cell leukemia, T and NK lymphoma [41, 42]. However, our work and another report on hematopoietic tumors of B-ALL pediatric Colombian patients [26], describes an under-expression of CD38 in the group of patients with poor prognosis. Similar to our findings, Quijano et al. [26] reported that higher FSC (larger size) was associated with patients with good prognostic. Expression of CD45, protein phosphatases that regulates various cellular functions and signaling pathways, has been reported low or negative in patients with chromosomal aberrations as t(12;21), t(4;11), hyperdiploidy, t(9;22), t(1;19) [43], which is consistent with our finding of reduced expression of this molecule in the group with poor prognostic features.

Regarding immunophenotypic markers associated with poor prognosis gene expression signature, patients with positive gene signature *ID1/ID3/IGJ* and poor outcome of the disease, showed over-expression of CD10 and CD20. It has been previously reported that over-expression of CD10 in patients with B-ALL is associated with the presence of poor prognosis genetic abnormalities as t(9; 22) [43]. Similarly, CD10 has been associated with worse prognosis in patients with melanoma [44] and high-grade malignancy and worse outcome in pancreatic cancer, gastric, colorectal [45], liver [46] and skin tumors [47]. On the other hand, CD20 expression has also been previously evaluated as a predictor of prognosis in patients with hematological malignancies [48–50]. Similar to our findings, other authors have reported that CD20 expression is associated with low survival [48] and therapies directed against CD20, such as Rituximab, have been implemented in hematological malignancies [49, 50]. However, controversial studies regarding the correlation between the expression of CD20 and prognosis have been published [51–53]. Therefore, it has been suggested that the prognostic significance of CD20 in B-ALL should be explored in other prospective studies with larger sample size.

With regards to the possible mechanism for low treatment response and poor survival of patients with gene expression signature *ID1/ID3/IGJ* and over-expression of CD10 or CD20, the *ID1* and *ID3* genes are transcription factors that inhibit of differentiation and have been reported as important participants in tumorigenic processes, tumor progression, angiogenesis, cellular migration, epithelial-mesenchymal transition and tumor cell self-renewal [23, 24, 54–64]. CD10, in turn, in addition to being a useful marker in cell differentiation to discriminate maturational stages in B-lineage, has also been described as playing a fundamental role in the extracellular microenvironment [44]. Several reports have shown that in various types of cancer, including hematopoietic tumors, CD10 has high tumorigenic activity and can promote tumor progression by regulating gene expression profiles related to cell proliferation, angiogenesis and apoptosis resistance [44, 65, 66]. Furthermore, CD10 promotes tumor stem cells proliferation and can regulate different intracellular signaling pathways that promote survival and adhesion such as PI3K-Akt, PTEN and adhesion through FAK [66]. Additionally, with its peptidase activity, CD10 modulates accumulation of peptides involved in cell proliferation and tumor progression in prostate, lung and pancreatic cancer [66]. In colorectal cancer, CD10 degrades Met-enkephalin, accelerating the tumor growth and liver metastasis [66]. Meanwhile, CD20 is a tetraspanin with ability to form calcium channels and regulate cell cycle progression and activation, differentiation and proliferation of B cells [67].

Overall, despite gene expression levels show high dispersion between patients and differences of ID1/ID3/IGJ expression between groups 1 and 3 classified based on immunophenotype is not too big, the high expression of inhibitors of differentiation (ID1 and ID3) together with the overexpression of the immaturity marker CD10 and aberrant expression of CD20, suggest that may there are active processes blocking differentiation, leading to maturation asynchronism that may confer more aggressive tumor characteristics and may lead to the activation of signaling pathways that contribute to resistance to chemotherapeutic treatment. These findings support the fact that there are differences in the prognosis of patients due to an underlying molecular signature (immunophenotype + gene expression) that could be playing an important role in the pathogenesis of the disease and provides unfavorable characteristics for clinical outcome.

This study has some limitations, which have to be pointed out. Due to this work represents only a small part of the Colombian population with B-ALL the results should be interpreted with caution since it is possible that factors other than difference of the expression level of our gene expression signature and its correlation wit phenotypic markers may influence the prognosis and outcome of patients. Additional studies including a larger series of patients are needed to obtain more significant differences and confirm the potential use of the *ID1/ID3/IGJ* as a prognostic marker. Despite the above limitations, this work will be the subject of ongoing studies.

These results support the utility of EuroFlow immunophenotype of B-ALL cells combined with the assessment of *ID1/ID3/IGJ* gene expression as a potential, independent prognostic indicator in B-ALL for both OS and EFS. Importantly, determining the expression of these elements could be used as a powerful yet simple and inexpensive prognostic tool. Thus, further screening and confirmation studies are required to validate our findings and determine whether ID1/ID3/IGJ measurements should become routine in B-ALL.

## Additional files

Additional file 1: Comparative analysis of mean fluorescence channels for the 19 markers included in the Euroflow panel and the parameters of light scattering (SSC and FSC) of leukemic cells from 42 B-ALL adult patients compared to three normal bone marrow samples. The box incorporates the middle quartiles; the line represents the median value: the whiskers indicate the minimum and maximum values. There is gain in expression of CD45, CD10, CD58, CD22, CD66, CD24, CD34, CD19 and FSC, SSC in B-ALL samples (B-ALL) as compared to normal bone marrow (Normal). There is loss in expression of CD38, CD117, CD9, TdT in B-ALL samples as compared to normal bone marrow. * *P* <0.05 with respect of pre-pre-B normal cells. (TIFF 910 kb)
Additional file 2: Characteristics of pronostic groups according to EuroFlow immunophenotype. (TIFF 1364 kb)
Additional file 3: Expression levels of immunophenotypic markers in the two groups formed at the extremes of heatmap (group 1 and group 3) with different clinical characteristics. No statistically significant differences were observed in the expression of CD20, CD58, CD66, CD117, CD34, CD33, SmIgM, CD13, Lambda, CD9, TdT, CD22, CD24, SSC. (TIFF 1519 kb)

## Abbreviations

B-ALL: B- Acute Lymphoblastic Leukemia
CR: Complete Remission
DFS: Disease Free Survival
FSC: Forward-Scatter
LAP: Leukemia Associated Phenotype
MFI: Mean Fluorescence Intensity
MRD: Minimal Residual Disease
SOP: Standard Operating Procedures
SSC: Side- Scatter
WBC: C White Blood Cell Count
WHO: World Health Organization
