# Supplementary figures and images for: Prognostic stratification improvement by integrating ID1/ID3/IGJ gene expression signature and immunophenotypic profile in adult patients with B-ALL
Source: J Exp Clin Cancer Res. 2017 Feb 28;36:37. doi: 10.1186/s13046-017-0506-4 (PMC5331651; doi:10.1186/s13046-017-0506-4)

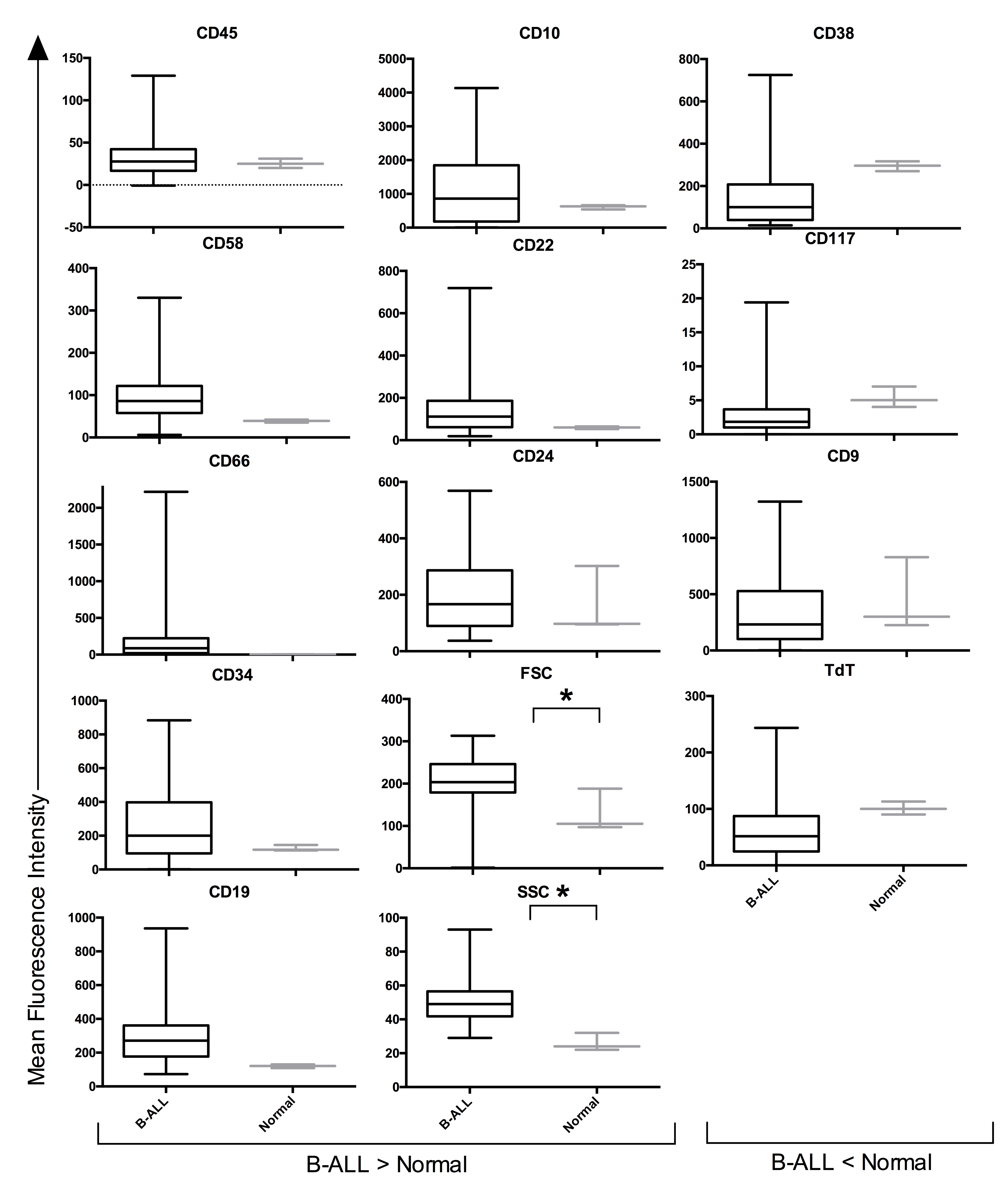

Supplement: Additional file 1: — Comparative analysis of mean fluorescence channels for the 19 markers included in the Euroflow panel and the parameters of light scattering (SSC and FSC) of leukemic cells from 42 B-ALL adult patients compared to three normal bone marrow samples. The box incorporates the middle quartiles; the line represents the median value: the whiskers indicate the minimum and maximum values. There is gain in expression of CD45, CD10, CD58, CD22, CD66, CD24, CD34, CD19 and FSC, SSC in B-ALL samples (B-ALL) as compared to normal bone marrow (Normal). There is loss in expression of CD38, CD117, CD9, TdT in B-ALL samples as compared to normal bone marrow. * P <0.05 with respect of pre-pre-B normal cells. (TIFF 910 kb) [file 13046_2017_506_MOESM1_ESM.tiff]

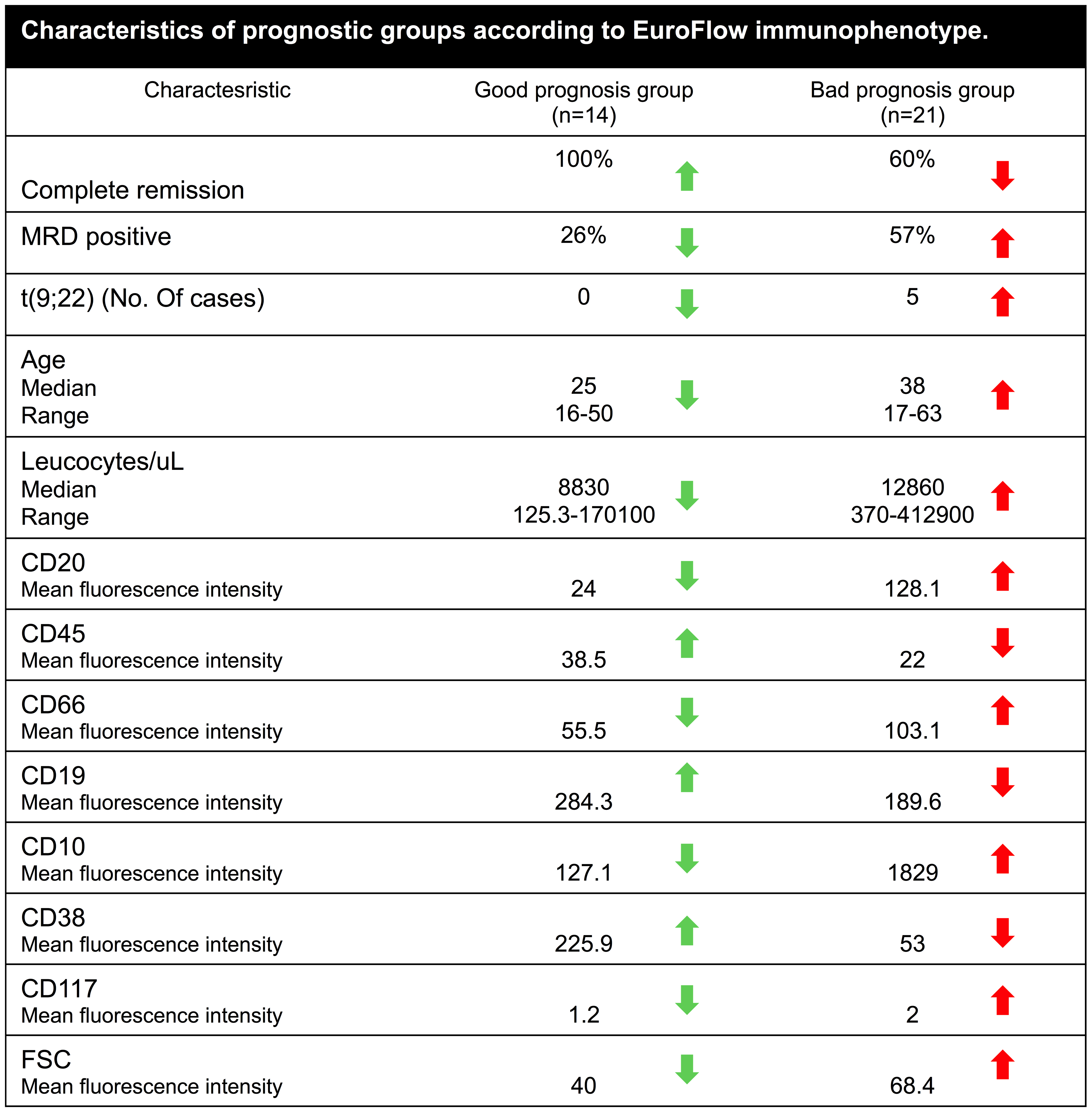

Supplement: Additional file 2: — Characteristics of pronostic groups according to EuroFlow immunophenotype. (TIFF 1364 kb) [file 13046_2017_506_MOESM2_ESM.tiff]

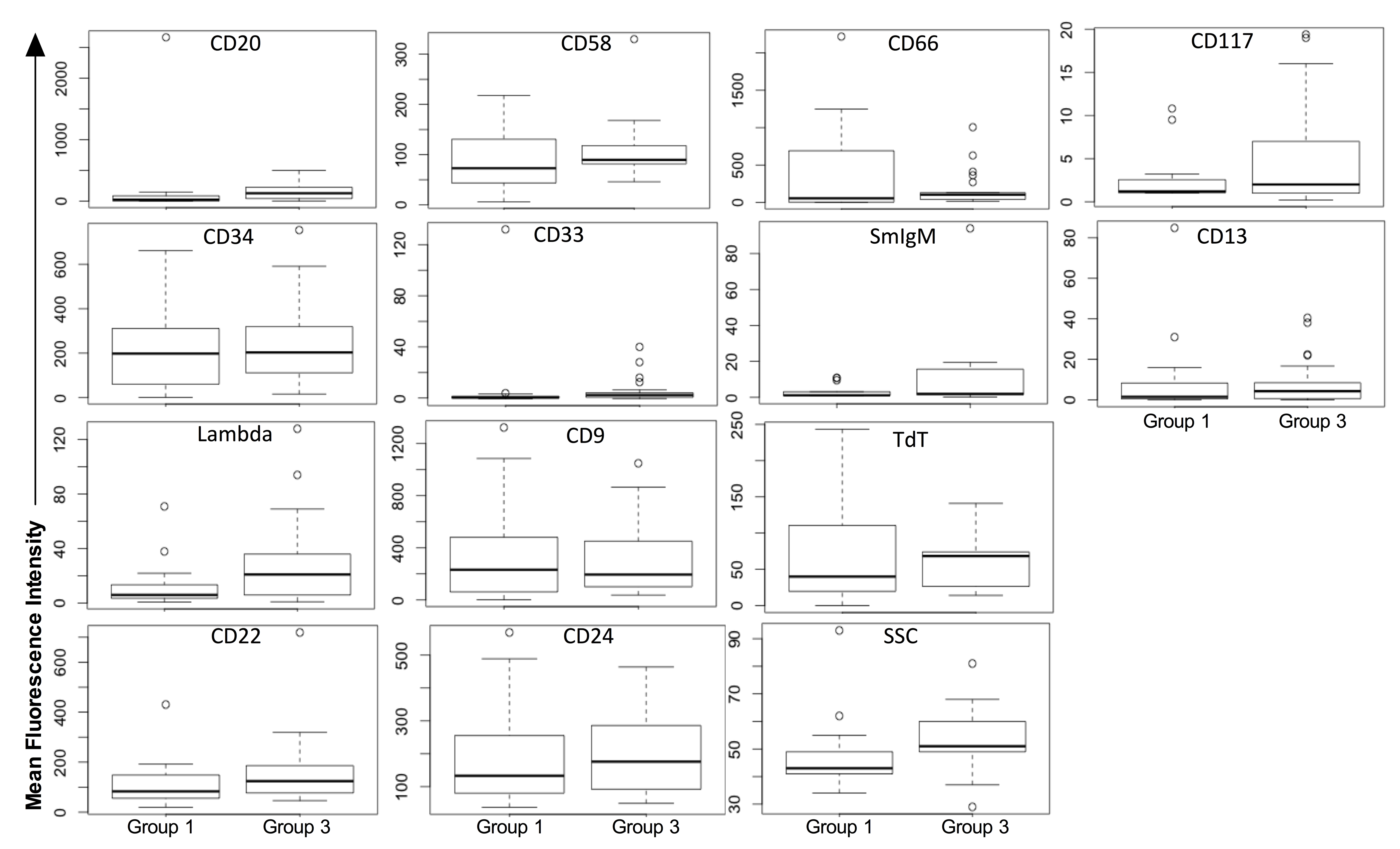

Supplement: Additional file 3: — Expression levels of immunophenotypic markers in the two groups formed at the extremes of heatmap (group 1 and group 3) with different clinical characteristics. No statistically significant differences were observed in the expression of CD20, CD58, CD66, CD117, CD34, CD33, SmIgM, CD13, Lambda, CD9, TdT, CD22, CD24, SSC. (TIFF 1519 kb) [file 13046_2017_506_MOESM3_ESM.tiff]
